# Nanoenviroments of the β-Subunit of L-Type Voltage-Gated Calcium Channels in Adult Cardiomyocytes

**DOI:** 10.3389/fcell.2021.724778

**Published:** 2022-01-03

**Authors:** Yiliam Cruz-Garcia, Katalin Barkovits, Michael Kohlhaas, Simone Pickel, Michelle Gulentz, Cornelia Heindl, Kathy Pfeiffer, Petra Eder-Negrin, Christoph Maack, Katrin Marcus, Michaela Kuhn, Erick Miranda-Laferte

**Affiliations:** ^1^ Institute of Physiology, University of Würzburg, Würzburg, Germany; ^2^ Medizinisches Proteom-Center, Medical Faculty, Ruhr-University Bochum, Bochum, Germany; ^3^ Medical Proteome Analysis, Center for Proteindiagnostics (PRODI), Ruhr-University Bochum, Bochum, Germany; ^4^ Comprehensive Heart Failure Center, University Hospital Würzburg, Würzburg, Germany; ^5^ Institut für Biologische Informationsprozesse, Molekular- und Zellphysiologie (IBI-1), Forschungszentrum Jülich, Jülich, Germany

**Keywords:** APEX-mediated proximity labeling, cardiomyocytes, protein-protein interaction, ryanodine receptor, β-subunit voltage-gated calcium channels, calcium-induced calcium release

## Abstract

In cardiomyocytes, Ca^2+^ influx through L-type voltage-gated calcium channels (LTCCs) following membrane depolarization regulates crucial Ca^2+^-dependent processes including duration and amplitude of the action potentials and excitation-contraction coupling. LTCCs are heteromultimeric proteins composed of the Ca_v_α_1_, Ca_v_β, Ca_v_α_2_δ and Ca_v_γ subunits. Here, using ascorbate peroxidase (APEX2)-mediated proximity labeling and quantitative proteomics, we identified 61 proteins in the nanoenvironments of Ca_v_β_2_ in cardiomyocytes. These proteins are involved in diverse cellular functions such as cellular trafficking, cardiac contraction, sarcomere organization and excitation-contraction coupling. Moreover, pull-down assays and co-immunoprecipitation analyses revealed that Ca_v_β_2_ interacts with the ryanodine receptor 2 (RyR2) in adult cardiomyocytes, probably coupling LTCCs and the RyR2 into a supramolecular complex at the dyads. This interaction is mediated by the Src-homology 3 domain of Ca_v_β_2_ and is necessary for an effective pacing frequency-dependent increase of the Ca^2+^-induced Ca^2+^ release mechanism in cardiomyocytes.

## Introduction

Ca^2+^ ions play a very important role as signal transducers in cardiomyocytes. In these cells, Ca^2+^ influx through L-type voltage-gated calcium channels (LTCCs) following membrane depolarization regulates crucial processes including duration and amplitude of the action potentials, excitation-contraction coupling and gene expression (Catterall, 2011). During cardiomyocyte contraction, the free cytosolic Ca^2+^ concentration increases from a resting level of ∼100 nM to ∼1 μM (Marks, 2013). This 10-fold difference is caused by Ca^2+^ currents through LTCCs located at the transverse tubules (t-tubules) in the dyadic junctions, which lead to the activation of the ryanodine receptors 2 (RyR2) in the sarcoplasmic reticulum (SR) membrane and the release of high Ca^2+^ amounts from this compartment. This process is termed Ca^2+^-induced Ca^2+^ release (CICR) and triggers sarcomeric contractions. Moreover, CICR plays a very important role in controlling the positive force-frequency relationship (FFR), an intrinsic regulatory mechanism of cardiac contractility. At positive FFR, a rise in the heart rate enhances CICR leading to a significant increase in the cardiac contractile force (Krishna et al., 2013).

Intracellular Ca^2+^ diffusion is limited by cytoplasmic buffers and membrane Ca^2+^ transporters that affect the time course and magnitude of the Ca^2+^ signal. In accordance, LTCC-mediated Ca^2+^ currents increase the intracellular Ca^2+^ levels only within few nanometers around the inner vestibule of the channel (Augustine et al., 2003). Consequently, most of the proteins directly regulated by the Ca^2+^ entering the cell through the LTCCs should be located in the LTCC nanoenvironments and possibly be arranged in macromolecular complexes with the channel. This supramolecular organization should relay the Ca^2+^ signal with high precision, speed and efficiency (Alberts, 2002) and define diverse LTCC subpopulations which specifically regulate particular LTCC-dependent mechanisms. However, the identity of most of the proteins located in the LTCC nanoenvironments and the mechanism targeting them to the channel vicinity remain largely unknown.

LTCCs are heteromultimeric proteins composed of a Ca_v_α_1_ pore-forming subunit and the Ca_v_β, Ca_v_α_2_δ and Ca_v_γ accessory subunits (Hofmann et al., 2014). Ca_v_β is a membrane-associated guanylate kinase (MAGUK) scaffolding protein. It is a cytosolic protein that binds with high affinity at an equimolar stoichiometry to the alpha interaction domain (AID) in the I–II loop of Ca_v_α_1_ (van Petegem et al., 2004). There are four non-allelic Ca_v_β isoforms (Ca_v_β_1_-β_4_), all of which consist of five domains including a conserved core that is composed of a Src-homology 3 (SH3) and a guanylate-kinase (GK)-like domain. These conserved domains are flanked by three regions that are highly variable in length and amino acid composition between the different isoforms: the N-terminus (NT), the C-terminus (CT) and the HOOK domain that separates the SH3 and GK domains. Of the four Ca_v_β isoforms, Ca_v_β_2_ seems to be the one predominantly expressed in cardiomyocytes (Meissner et al., 2011). Knockout mice lacking the expression of Ca_v_β_2_ die at early embryonic stages due to impaired cardiac development and contractile function (Weissgerber et al., 2006). Moreover, mutations in the human Ca_v_β_2_ gene have been associated with the Brugada syndrome, characterized by cardiac arrhythmias and sudden death (Simms, 2014). There are five splice variants of Ca_v_β_2_ (Ca_v_β_2a_, Ca_v_β_2b_, Ca_v_β_2c_, Ca_v_β_2d_ and Ca_v_β_2e_), which differ only in the N-terminal region and confer different electrophysiological properties when coexpressed with the channel (Link et al., 2009).

For many years, Ca_v_β was only considered as a regulator of LTCC trafficking and activity. Recently, however, Ca_v_β has been directly linked to diverse cellular processes including cellular trafficking, endocytosis and gene expression (Hofmann et al., 2015). Furthermore, given its cytosolic character and high affinity for the LTCCs, we envision that this protein could play a very important role as a molecular scaffold by recruiting and organizing protein complexes in the LTCCs nano-environments in cardiomyocytes.

In this work, we combined ascorbate peroxidase (APEX2)-mediated proximity biotinylation and quantitative proteomics to identify proteins within the Ca_v_β_2_ nanoenvironments in cardiomyocytes. This approach identified 61 proteins in the nanoenvironments of Ca_v_β_2_. Moreover, it showed that in the dyads of these cells, Ca_v_β_2_ and the RyR2 are coupled into a supramolecular complex mediated by the SH3 domain of Ca_v_β_2_. Such complex is needed for the pacing frequency-dependent increase of CICR and therefore for an adequate cardiac contractility at high stimulation frequencies.

## Materials and Methods

### Production of Recombinant Adenovirus

To generate the plasmid pENTR3C Ca_v_β_2b_-V5-APEX2, a cDNA fragment containing the APEX A134P (APEX2) open reading frame was obtained by PCR amplification from the vector pTRC-APEX2 (Lam et al., 2015). The cDNA encoding full rat Ca_v_β_2b_ (accession number Q8VGC3-5) was amplified by PCR from the pcDNA3.1-Ca_v_β_2b_ vector. The Ca_v_β_2b_-derived PCR fragment was cloned in frame with the APEX2 PCR fragment into the pENTR3C vector (Thermo Fisher Scientific) using conventional molecular biology methods. To prepare the construct Ca_v_β_2b_-SH3-V5, the SH3 domain of the rat Ca_v_β_2b_ subunit (residues 25–137) was amplified by PCR from the pcDNA3.1-Ca_v_β_2b_ vector with the forward primer containing as overhang the cDNA sequence of the V5 tag. The resulting PCR fragment was cloned into the pENTR3C vector using conventional molecular biology methods. Adenoviral vectors for the overexpression of proteins Ca_v_β_2b_-V5-APEX2 and Ca_v_β_2b_-SH3-V5 were generated by homologous DNA recombination between each pENTR3C plasmid and the pAd/CMV/V5-Dest vector, using the pAd/CMV/V5-DEST™ Gateway® Vectors (Thermo Fisher Scientific) according to the manufacturer’s instructions. To generate a virus for the transduction of control cells in the electrophysiological experiments and Ca^2+^ transient measurements, the incompatible overhangs of the empty linear pENTR/U6 vector (Thermo Fisher Scientific), were converted to blunt-ended DNA using the Quick Blunting™ Kit (New England Biolabs). The vector was then circularized by blunt-end ligation with standard molecular biology methods and used to produce the control virus by homologous DNA recombination with the pAd/BLOCK-iT™-DEST (Thermo Fisher Scientific) plasmid. All the resulting recombinant adenoviral plasmids were linearized with the PacI restriction enzyme and transfected into HEK-293A cells (Thermo Fisher Scientific) using the X-tremeGENE™ HP DNA Transfection Reagent (Roche), following the manufacturer’s instructions. Cells were harvested 14 days after transfection, when 70–80% cytopathic effect was visible, and subjected to three freeze-thaw cycles. The supernatants were collected by centrifugation and viral titers were determined by plaque assay using Avicel overlay medium, as previously described (Baer and Kehn-Hall, 2014).

### Isolation and Adenoviral Transduction of Adult Rat Cardiomyocytes

For each adult rat cardiomyocyte (ARC) isolation, one Wistar rat (250–300 g, Charles River Laboratories) was euthanized and the heart was surgically excised, washed with buffer A (in mM: NaCl 113, NaHCO_3_ 12, HEPES 10, KH_2_PO_4_ 0.6, Mg_2_SO_4_ × 7H_2_O 1.22, KHCO_3_ 10, Na_2_HPO_4_ 0.6, KCl 4.69, phenol red 0.03, glucose 5.5, taurine 30, 2.3-butanedione monoxime 10, pH 7.46 adjusted with NaOH) and placed in a Langendorff perfusion apparatus via cannulation of the aorta. The heart was flushed blood-free for 4 min with buffer A and perfused during 7 min with recirculating enzyme buffer (20 ml Buffer A supplemented with 1.44 mg liberase (Roche), 5.6 μg trypsin (Sigma Aldrich), 12.5 μM CaCl_2_). Then, the ventricles were collected, minced, and the cells were dissociated by gentle shaking in 5 ml buffer A and 5 ml stop buffer 1 (9 ml buffer A, 1 ml fetal calf serum (FCS), 12.5 μM CaCl_2_), allowing them to settle for 10 min at RT. The resulting pellet was resuspended in stop buffer 2 (28.5 ml buffer A, 1.5 ml FCS, 12.5 μM CaCl_2_) and subjected to several Ca^2+^ adaptation steps of 4 min each, increasing the Ca^2+^ concentration in buffer A stepwise (0.05 mM, 0.1 mM, 0.2 mM, 0.5 mM, 1 mM final). After the last step, the cells were resuspended in modified M199 medium (870 nM insulin, 65 nM transferrin, 29 nM Na-selenite, antibiotics (100 U/ml penicillin, 100 μg/ml streptomycin) with additional FCS 5% (v/v). 1 × 10^5^ cells were plated in laminin-coated 6-well plates or onto glass coverslips. After 3 h of incubation, the medium was replaced with serum-free modified M199 medium. For viral transduction, ARCs were exposed to 5–75 plaque-forming units (pfu)/cell of adenovirus during 4 h at 37°C and then medium was replaced with modified M199 medium and the cells incubated at 37°C for 24 h.

### RT-PCR

The expression of the different Ca_v_β_2_ splice variants in ARCs was determined by RT-PCR. After isolation of total RNA from 1 × 10^5^ cells using a TRIzol^™^-based method, RT-PCR was performed using the following forward primers: 5′-ATG​CAG​TGC​TGC​GGG​CTG​G-3′ for Ca_v_β_2a_, 5′-ATG​CTT​GAC​AGG​CAG​TTG​GTG​TCT​TC-3′ for Ca_v_β_2b_, 5′-ATG​GAC​CAG​GCG​AGT​GGA​CTG​G-3′ for Ca_v_β_2c_, 5′-TGA​TGA​CAT​CTG​TAT​CTG​GCA​AAC​CAG-3′ for Ca_v_β_2d_, 5′-ATG​AAG​GCC​ACC​TGG​ATC​AGG​C-3′ for Ca_v_β_2e_ and a common antisense primer 5′-TCT​TTA​ACC​AGC​CGT​CCT​ATC​CAC​C-3′. For the detection of GAPDH the forward primer: 5′-GAA​TGG​GAA​GCT​GGT​CAT​CAA​CG-3′ and the reverse primer: 5′-TGC​ATT​GCT​GAC​AAT​CTT​GAG​GGA​G-3 were used.

### Antibody Preparation and Validation

To prepare an antibody specifically recognizing Ca_v_β_2b_, the DNA encoding the variable N-terminus (NT_v_) from rat Ca_v_β_2b_ (residues 1–17; accession number Q8VGC3-5) was amplified by PCR from the pcDNA3.1--Ca_v_β_2b_ vector and cloned into the PGEX-6P-1 vector to fuse a glutathione S-transferase (GST) moiety at the N-terminus of Ca_v_β_2b_-NT_v_. The resulting GST-Ca_v_β_2b_-NT_v_ recombinant protein was expressed in the *E. coli* BL21 (DE3) host strain by a 3-h induction with 0.5 mM isopropyl-β-D-thiogalactopyranosid (IPTG). Cells were harvested by centrifugation and stored at −80°C until used. Before protein purification, the cells were resuspended in PBS pH 7.4, lysed by sonication. The GST-Ca_v_β_2b_-NT_v_ recombinant protein was purified by affinity chromatography using a GST HiTrap™ column (GE Healthcare) followed by size exclusion chromatography. The recombinant protein was concentrated by ultrafiltration using an Amicon® Ultra centrifugal filter (Merck). Protein concentration was measured spectrophotometrically at 280 nm. The GST-Ca_v_β_2b_-NT_v_ recombinant protein was used as antigen to immunized rabbits for the production of the anti-Ca_v_β_2b_ antibody. The serum collected from rabbits 8 weeks after the antigen immunization were used to purify the anti-Ca_v_β_2b_ antibody by affinity chromatography using maltose-coated beads previously incubated with the MBP-Ca_v_β_2b_-NT_v_ fusion protein. This protein was prepared after cloning the Ca_v_β_2b_-NT_v_ fragment into the pMAL5 vector (New England Biolabs) and following the same protein purification steps described for the GST-Ca_v_β_2b_-NT_v_ recombinant protein, but using a MBPtrap™ column (GE Healthcare) to perform the affinity chromatography step.

To validate the specificity of the anti-Ca_v_β_2b_ antibody, HEK-293 cells (Thermo Fisher Scientific) were grown in Dulbecco’s modified Eagle’s medium supplemented with 10% fetal bovine serum and L-glutamine (2 mM) and incubated in a 5% CO_2_-humidified atmosphere. Cells (1 × 10^5^) were transiently transfected using X-tremeGENE™ HP DNA Transfection Reagent (Roche) according to the manufacturer’s instructions with the corresponding plasmids encoding the five different Ca_v_β_2_ splice variants. Twenty-four hours after transfection cells were lysed on ice using cell lysis buffer (CLB; 20 mM Tris, 150 mM NaCl, 1% NP-40, pH 7.5) supplemented with 1× protease inhibitor cocktail and 1 mM EDTA and centrifuged at 16,000 g for 15 min at 4°C. The supernatants were collected and stored at −80°C until used in western blot analyses.

### Biotin Labeling and Streptavidin Bead Enrichment of Biotinylated Proteins

The biotin labeling was performed as previously described (Hung et al., 2016). ARCs (1 × 10^5^ cells) were transduced with Ca_v_β_2b_-V5-APEX2 adenovirus. Twenty-four hours after transduction, the cells were pre-incubated with 0.5 mM biotin-phenol in culture medium for 30 min at 37°C, followed by a 1-min incubation with H_2_O_2_ at a final concentration of 1 mM. To stop the reaction, cells were washed with quenching solution (5 mM Trolox, 10 mM sodium azide and 10 mM sodium ascorbate in PBS). Cells were lysed on ice using CLB supplemented with 1× protease inhibitor cocktail and 1 mM EDTA and centrifuged at 16,000 g for 15 min at 4°C. From each reaction, 200 μg of protein were incubated with 50 μl of streptavidin-coated magnetic bead slurry for 3 h at 4°C. After washing the streptavidin beads with CLB, the biotinylated proteins were eluted with 2× Laemmli buffer and boiled for 5 min.

### Sample Preparation and Tryptic Digestion for Mass Spectrometry

The samples eluted from the streptavidin-coated beads after enrichment of biotinylated proteins were loaded on a 12% polyacrylamide Bis-Tris gel and the electrophoresis was performed at 25 V for 10 min. The protein bands were excised, hashed and destained by incubating the gel pieces three times with 100 mM ammonium bicarbonate for 10 min, alternated with three 10 min incubations with a 1:1 (v/v) mixture of 100 mM ammonium bicarbonate and 100% acetonitrile. Digestion was performed overnight at 37°C with 30 µl of trypsin solution (0.006 µg/µl, Serva). The peptides were eluted by incubating two times during 10 min with 40 μl of a 1:1 (v/v) solution containing 100% acetonitrile and 0.1% (v/v) trifluoroacetic acid (TFA). Samples were dried and resuspended in 15 μl of 0.1% (v/v) TFA. The peptide concentration was determined as previously described (Plum et al., 2013) and 200 ng per sample were used for MS analysis.

### Identification and Quantification of Proteins by Mass Spectrometry

Nano-HPLC-MS/MS was performed as previously described (Maerkens et al., 2016) by means of LC-MS/MS on an UltiMate 3000 RSLCnano system coupled online to an LTQ Orbitrap Elite mass spectrometer (both from Thermo Fisher Scientific). For protein identification via database searches, the raw files were analyzed with the Proteom Discoverer software (version. 1.4.1.14) (Thermo Fisher Scientific) using the Mascot search algorithm (version 2.5) (Matrix Science Ltd.) and searching against the UniProtKB/Swiss-Prot database using rat taxonomy (released 2018_11, 36075 sequences entries in the whole database). The database search was performed with the following parameters: mass tolerance 5 ppm for precursor and 0.4 Da for fragment ions; 1 missed cleavage; methionine oxidation and biotinylation as variable modifications; false discovery rate (FDR) <1%. We accepted all proteins identified in MS analyses by more than two different peptides. Label-free quantification of proteins was performed using MaxQuant (version 1.5.3.8, Max-Planck-Institute Martinsried) (Cox and Mann, 2008) and the raw data processing as previously described (Keilhauer et al., 2015). Only unique peptides were used for quantification.

### Analysis of the Mass Spectrometry Data

The analysis of the label-free quantification (LFQ) data was performed using the Perseus software (version 1.6.5.0, Max-Planck-Institute Martinsried). The “proteingroups.txt” file generated by MaxQuant was loaded into Perseus and LFQ intensities were transformed to a logarithmic scale. Only the proteins containing LFQ intensity values in all the three replicates of the pull-down of Ca_v_β_2b_-V5-APEX2-biotinylated proteins were used for the analysis. The resulting differences between the means of the logarithmized LFQ intensities for each protein in the samples from H_2_O_2_-treated and untreated Ca_v_β_2b_-V5-APEX2-expressing cells [log_2_(+H_2_O_2_/−H_2_O_2_)] were plotted in a volcano plot against the negative logarithmized p values. The classification of proteins as constituents of the Ca_v_β_2b_ nano-environments was based on a cutoff value separating enriched proteins from background binders. To determine the cutoff value, the standard deviation (SD) between the means of logarithmized LFQ intensity values of the samples from Ca_v_β_2b_-V5-APEX2-expressing cells treated with H_2_O_2_ and those from untreated cells (negative control) was calculated. Only proteins with mean differences, between H_2_O_2_ treated samples and the negative controls, higher than one SD were accepted at *p* < 0.01. This LQF analysis was performed as previously reported (Keilhauer et al., 2015).

The mass spectrometry proteomics data have been deposited to the ProteomeXchange Consortium via the PRIDE (Perez-Riverol et al., 2019) partner repository with the dataset identifier PXD021375 and 10.6019/PXD021375.

### Expression and Purification of Recombinant Proteins

To produce the recombinant proteins used for pull-down assays, the cDNA from the full rat Ca_v_β_2b_ and its NT (residues 1–24), SH3 (residues 25–137), HOOK (residues 138–225) and CT (residues 424–605) domains were amplified by PCR. Each specific forward primer contained as overhang the cDNA sequence encoding the Twin-Strep-tag peptide (WSHPQFEKGGGSGGGSGGSAWSHPQFEK). The resulting PCR fragments were cloned in frame into the pGEX-6P-1 vector (Sigma-Aldrich) to fuse a GST moiety at the N-terminus of the resulting proteins. In parallel, the construct pRSET B-His-Twin-Strep-Ca_v_β_2b_ was generated by cloning in frame into the pRSET B vector (Invitrogen) the PCR fragment corresponding to the Ca_v_β_2b_. This construct carries at its 5’ end the sequence coding for a polyhistidine tag, followed by the Strep tag. The GST fusion proteins were purified as described above for the GST-Ca_v_β_2b_-NT_v_ protein. The purification of the His-Twin-Strep-Ca_v_β_2b_ protein was performed mostly as described for the GST-Ca_v_β_2b_-NT_v_ protein, but the cells were resuspended using 20 mM imidazole in PBS, pH 7.4 and the affinity chromatography was performed using a HisTrap™ column (GE Healthcare).

### Strep-Tag Pull-Down

Strep-Tactin^®^-coated beads (MagStrep type3 XT, IBA) were incubated with equal molar amounts of the GST-Twin-Strep-tagged proteins (0.2 nmol) for 1 h at 4°C in a final volume of 250 μl of CLB. Then the beads were washed with chilled CLB. Mouse hearts were powdered using a Mikro-Dismembrator U (Sartorious). Proteins were extracted with CLB supplemented with 1× protease inhibitor cocktail and 1 mM EDTA by vortexing on ice. Insoluble components were removed by centrifugation at 16,000 g for 15 min at 4°C. The lysates were incubated for 3 h at 4°C in a rotating mixer with the GST-Twin-Strep-tagged proteins previously bound to the Strep-Tactin^®^-coated beads. Following incubation, samples were washed using CLB, eluted with 2× Laemmli buffer and boiled for 5 min at 95°C or incubated at RT for 45 min and then resolved by SDS-PAGE and analyzed by western blot or mass spectrometry.

### Co-Immunoprecipitation

Mouse hearts were lysed as described in the previous section. The lysates were incubated overnight at 4°C with Protein G beads (Dynabeads Protein G, Invitrogen) and 8 μg of either anti-Ca_v_β_2b_ antibody or normal mouse IgG as a negative control. Then the beads were washed with CLB. Elution was performed by adding 2× Laemmli buffer to the beads, which were then incubated for 45 min at RT. The interaction with RyR2 was analyzed by western blotting.

### Western Blots

Forty micrograms of total protein lysates were resolved by SDS-PAGE, transferred onto nitrocellulose membranes and blocked with BSA. The membranes were subsequently incubated overnight at 4°C in blocking buffer with the corresponding primary antibody: Ca_v_β_2b_ (1:250, homemade), Ca_v_β_2_ (1:1,000, Novus Biologicals, NBP1 86680), Ca_v_1.2 (1:500, Alomone Labs, ACC-003), RyR2 (1:1,000, Thermo Fisher Scientific, MA3-916), V5 epitope tag (1:1,000, Cell Signaling, #13203), SERCA2a (1:1,000, Thermo Fisher Scientific, MA3-919) and GAPDH (1:5,000, Cell Signaling, #2118). Afterwards, the membranes were washed with TBS-Tween and incubated with anti-rabbit IgG or anti-mouse IgG secondary antibodies conjugated to horseradish peroxidase (HRP) for 1 h at RT. For the biotinylated proteins, the membranes were incubated with streptavidin-HRP conjugate (1: 2,000, Thermo Fisher Scientific) during 2 h at RT. After washing, all the membranes were developed using the PierceTM ECL Western Blotting Substrate (Thermo Fisher Scientific).

### Immunocytochemistry

ARCs (1 × 10^5^ cells) were plated on laminin-coated glass coverslips. After 3 h of incubation, the cells were washed and fixed in 4% paraformaldehyde at RT. Fixed cells were permeabilized with 0.2% Triton-X100, washed again and blocked with 5% normal goat serum (NGS). Cells were then rinsed and incubated overnight at 4°C with the corresponding primary antibody: Ca_v_β_2b_ (1:250, homemade), Ca_v_β_2_ (1:1,000, Novus Biologicals, NBP1 86680), V5 epitope tag (1:400, Cell Signaling, #13203), α-actinin 2 (1:250, Sigma Aldrich, A7811) and RyR2 (1:400, Thermo Fisher Scientific, MA3-916) diluted in 1.5% NGS. For confocal imaging, the coverslips were incubated, after washing, for 2 h at RT with the corresponding mouse or rabbit secondary antibodies conjugated to Alexa Fluor 488 or Alexa Fluor 633, respectively. Finally, cells were washed and mounted on glass slides using DAPI mounting medium (Dianova). Confocal microscopy images were acquired on a Leica inverted confocal microscope using a 60× oil-immersion objective and under the same high-voltage gain settings. Excitation was performed at 405 nm for DAPI, 488 nm for Alexa Fluor 488 and 633 nm for Alexa Fluor 633. The degree of co-localization between Ca_v_β_2b_ and RyR2 or α-actinin 2 was quantified by calculating the Manders coefficient using the JACoP plugin (Bolte and Cordelières, 2006) embedded in the ImageJ 1.44p software (National Institutes of Health, Bethesda) (Schneider et al., 2012).

### Electrophysiology

Calcium currents were recorded in whole-cell voltage-clamp mode (EPC-10 amplifier, Patchmaster software, HEKA Elektronik). Glass pipettes were pulled to 2–4 MΩ resistance and filled with internal solution (in mM: CsCl 125, HEPES 20, Mg-ATP 5, pH 7.2 adjusted with CsOH). ARCs were perfused with K^+^-free external solution (in mM: NaCl 140, CsCl 4, MgCl_2_ 1, HEPES 5, glucose 10, CaCl_2_ 1.8, pH 7.4 adjusted with NaOH). Current-voltage relationships were established using a voltage step protocol from −40 to + 50 mV, 200 ms, in 10-mV increments at 0.4 Hz, with a holding potential set to −70 mV. Data were acquired with Patchmaster and analyzed using the Fitmaster software (HEKA Elektronik) and Microsoft Excel.

### Calcium Measurement

ARCs (1 × 10^5^ cells) were cultured on laminin-coated cover slides and loaded for 20 min at RT with 1 μM of Fluo-4AM (Thermo Fisher Scientific) in a Ca^2+^-free normal Tyrode’s solution (in mM: NaCl 130, KCl 5, MgCl_2_ 1, HEPES 10, ascorbic acid 0.3, pyruvate 2, glucose 10, pH 7.4). Ca^2+^ measurements were performed using a customer-modified Myocyte Calcium Photometry and Contractility System from IonOptix. ARCs were sequentially paced at 0.5, 1.0, 2.0, 3.0 and 4.0 Hz while perfused with normal Tyrode’s solution containing 1.8 mM Ca^2+^. The whole experiment was conducted at 37°C. The dye was excited at 480 nm and the emitted fluorescence was measured at 535 nm. Fluorescence was recorded for 1 min and the means of ten Ca^2+^ transients for each stimulation frequency were used for the analysis (Ionwizard 6.3 software, IonOptix). SR Ca^2+^ content was estimated by rapid application of a caffeine pulse, pacing the ARCs at 1.0 Hz for 1 min to establish steady state contractions. Then, the pacing was paused and SR Ca^2+^ release was induced by fast perfusion of normal Tyrode’s solution supplemented with 1.8 mM Ca^2+^ and 10 mM caffeine.

### Statistical Analysis

All data are presented as the mean ± SEM. Statistical analyses were performed using the two-tailed unpaired Student’s *t*-test, one or two-way ANOVA as appropriate. The analyses were performed with GraphPad Prism version 7 (GraphPad Software Inc.) and Microsoft Excel.

## Results

### Ca_v_β_2b_-APEX2 Expression in Adult Rat Cardiomyocytes

**FIGURE 1:**
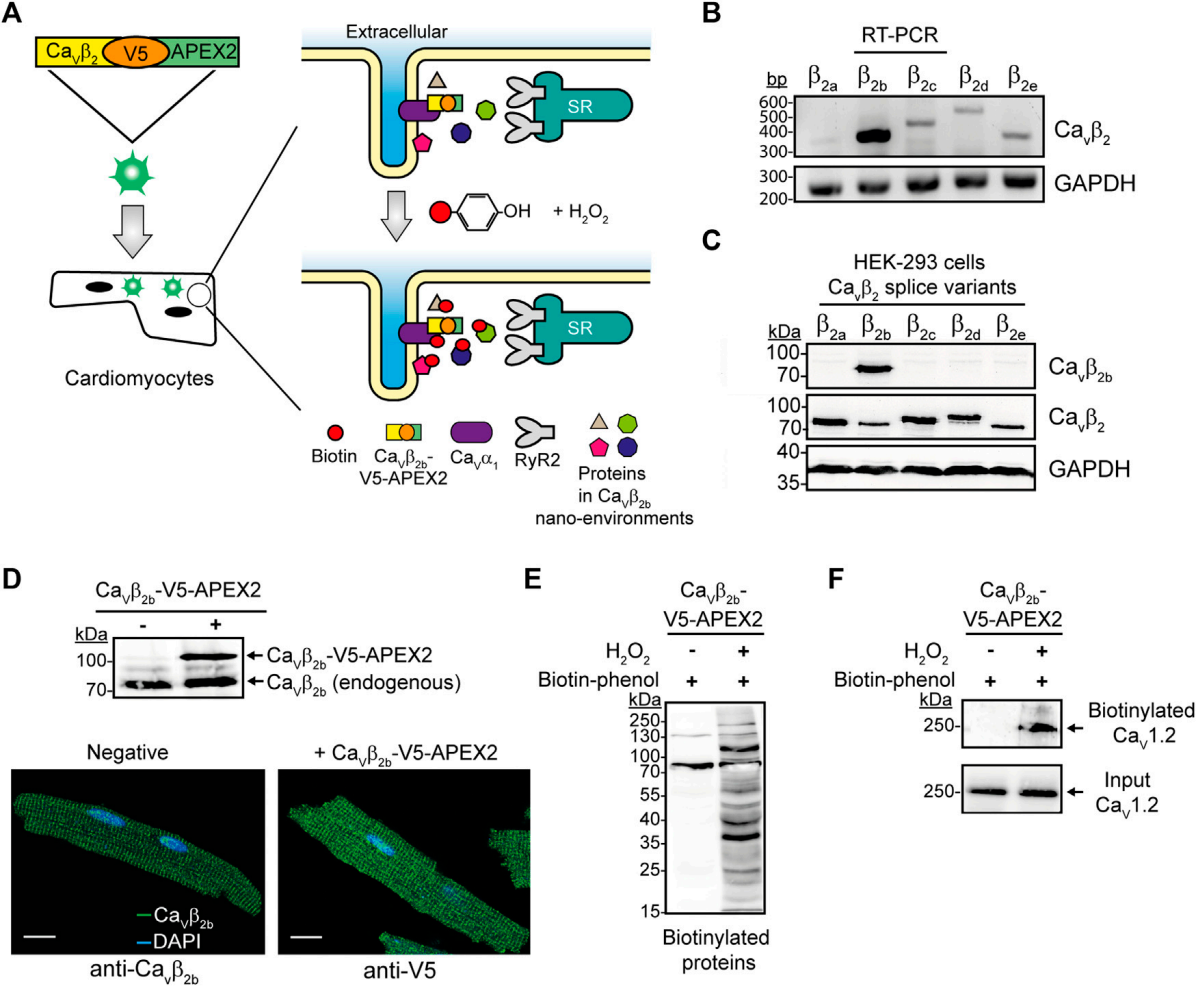
APEX2-mediated proximity biotinylation for the identification of proteins in the Ca_v_β_2b_ nanoenvironments in cardiomyocytes. **(A)** Schematic representation of the APEX2-mediated proximity biotinylation workflow. ARCs were transduced with the construct Ca_v_β_2b_-V5-APEX2 using an adenoviral vector. APEX2 fused to Ca_v_β_2b_ enables the biotin labeling of proteins located within a 20-nm radius of Ca_v_β_2b_ when the substrates biotin-phenol and H_2_O_2_ are present. Biotinylated proteins are further enriched by streptavidin-coated beads through affinity purification and bound proteins are analyzed by mass spectrometry. **(B)** RT-PCR analysis of the expression pattern of the five Ca_v_β_2_ splice variants (Ca_v_β_2a_-Ca_v_β_2e_) in ARCs. GAPDH was used as control. **(C)** Western blot analyses to validate the specificity of the anti-Ca_v_β_2b_ antibody using lysates from HEK-293 cells overexpressing the different Ca_v_β_2_ splice variants. A commercial anti-Ca_v_β_2_ and an anti-GAPDH antibodies were used as expression and loading controls, respectively. **(D)** Western blot analysis of Ca_v_β_2b_-V5-APEX2 expression in ARCs **(i)** and confocal fluorescence images of ARCs expressing or not Ca_v_β_2b_-V5-APEX2 and visualized using anti-Ca_v_β_2b_ and anti-V5 antibodies to recognize the endogenous and the recombinant Ca_v_β_2b_ proteins, respectively **(ii)**. Scale bar represents 15 μm. **(E)** Streptavidin blot analysis of endogenous proteins biotinylated by Ca_v_β_2b_-V5-APEX2 in ARCs. **(F)** Biotinylation of Ca_v_1.2 by Ca_v_β_2b_-V5-APEX2. Western blot analysis using an anti-Ca_v_1.2 antibody after streptavidin-mediated enrichment of biotinylated proteins. All the data shown are representative of three independent experiments. APEX2, ascorbate peroxidase; ARCs, adult rat cardiomyocytes; RyR2, ryanodine receptor 2; bp, base pairs.

Most of the cellular protein-protein interactions are transient, have a relatively low affinity or are disrupted after cell lysis (Perkins et al., 2010). APEX2-mediated proximity biotinylation in living cells combined with quantitative proteomics has emerged as a powerful method to map protein networks (Martell et al., 2012; Rhee et al., 2013). The treatment with biotin-phenol and H_2_O_2_ of cells expressing an engineered ascorbate peroxidase APEX2, results in the covalent labeling of proteins located within a 20-nm radius of each APEX2 molecule (Martell et al., 2012; Rhee et al., 2013). Since Ca_v_β_2_ is the predominant Ca_v_β isoform expressed in the cardiomyocytes (Weissgerber et al., 2006; Simms, 2014), the fusion of APEX2 to Ca_v_β_2_ could offer a means to detect proteins in the nano-environments of Ca_v_β_2_ and LTCCs in cardiomyocytes (Figure 1A).

In this study, we first corroborated the expression pattern of the five Ca_v_β_2_ splice variants (Ca_v_β_2a_-Ca_v_β_2e_) in cultured ARCs. RT-PCR analyses confirmed that Ca_v_β_2b_ is the predominant splice variant and that the other ones are detected at very low levels (Figure 1B) as also ocurrs in neonatal rat cardiomyocytes (Pickel et al., 2021). In order to detect the expression of protein Ca_v_β_2b_ in cardiomyocytes, we produced an antibody that specifically recognizes and targets the N-terminus of this Ca_v_β_2_ splice variant. Western blot analyses using cell lysates from HEK-293 cells overexpressing each Ca_v_β_2_ splice variant confirmed the high specificity of this antibody to Ca_v_β_2b_ (Figure 1C).

The C-terminus of Ca_v_β is predicted by circular dichroism to be intrinsically unstructured and its structure was not resolved in the crystallographic study of the protein (van Petegem et al., 2004). Moreover, neither the localization nor the functional properties of Ca_v_β as an LTCC regulator were affected by the fusion of diverse proteins to the C-terminus of Ca_v_β (Colecraft et al., 2002; Miranda-Laferte et al., 2014; Stolting et al., 2015). Therefore, we fused APEX2 to the C-terminus of a V5 epitope-tagged Ca_v_β_2b_ and transduced ARCs with this construct using an adenoviral vector (Figure 1A). As expected, the expression of a ∼70-kDa band corresponding to the endogenous Ca_v_β_2b_ was detected in non-transduced and Ca_v_β_2b_-V5-APEX2-expressing cardiomyocytes by western blot analysis using the anti-Ca_v_β_2b_ antibody. Additionally, a ∼100-kDa band corresponding to the expression of the Ca_v_β_2b_-V5-APEX2 construct was also detected in the cells transduced with the virus (Figure 1Di). Immunocytochemistry analyses of non-transduced and Ca_v_β_2b_-V5-APEX2-expressing cells, using the anti-Ca_v_β_2b_ antibody and an anti-V5 antibody, respectively, showed a similar fluorescence pattern for the endogenous Ca_v_β_2b_ and the overexpressed Ca_v_β_2b_-V5-APEX2 fusion protein (Figure 1Dii), demonstrating that the exogenous Ca_v_β_2b_-V5-APEX2 is not mislocalized in the cardiomyocytes.

Treatment of Ca_v_β_2b_-V5-APEX2-expressing ARCs with biotin-phenol and H_2_O_2_ induced extensive protein biotinylation, whereas almost no biotinylation was detected when H_2_O_2_ was omitted (Figure 1E). In agreement with the fact that Ca_v_1.2 is the main binding partner of Ca_v_β_2_ in cardiomyocytes, we observed a robust biotinylation of this protein in pull-down assays using streptavidin-coated beads and performed with lysates of Ca_v_β_2b_-V5-APEX2-expressing cells treated with biotin-phenol and H_2_O_2_ (Figure 1F). This demonstrates that the Ca_v_β_2b_-V5-APEX2 fusion protein mediates the biotin labeling of neighboring proteins in living cardiomyocytes.

### Ascorbate Peroxidase-Mediated Proximity Biotinylation Allowed the Identification of Multiple Proteins in the Ca_v_β_2b_ Nanoenvironments

**FIGURE 2:**
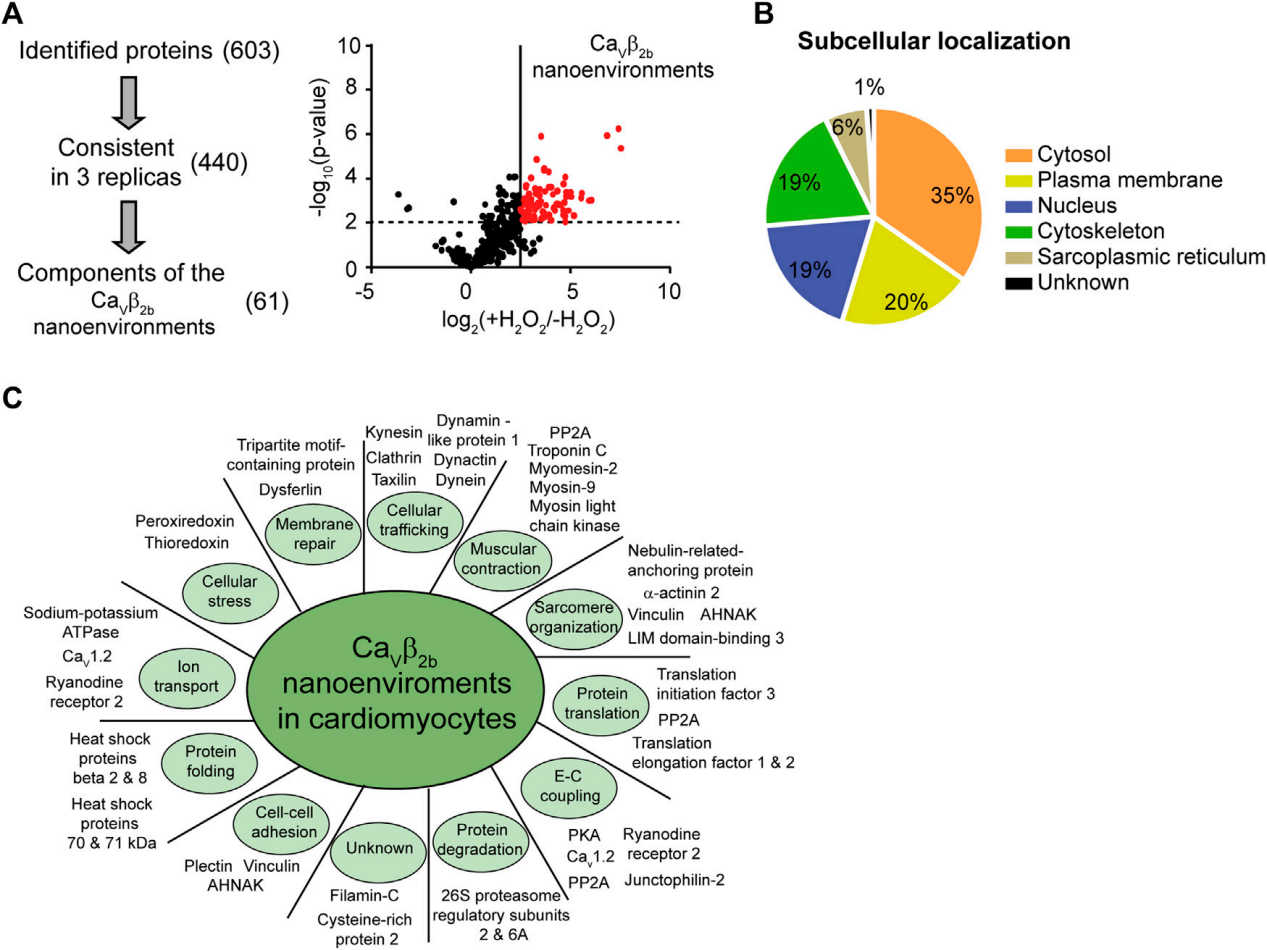
Identification of proteins in Ca_v_β_2b_ nanoenvironments by mass spectrometry. **(A)** Three staged filters (identification, consistency and exclusion) were used to extract high-confidence protein constituents of the Ca_v_β_2b_ nanoenvironments. **(i)** The identification filter included the proteins identified with more than two different peptides and at a false discovery rate of 1%. The consistency filter included the proteins that were identified in all the replicates. Components of Ca_v_β_2b_ nanoenvironments were accepted after an exclusion filter including only the proteins enriched by more than one standard deviation at *p* < 0.01. **(ii)** Differences between the logarithmized means of protein intensities in Ca_v_β_2b_-V5-APEX2 versus control samples are plotted against the −log_10_ (*p*-value) in a volcano plot. Experiments were performed in triplicate. Statistical significance was set at *p* < 0.01, corresponding to −log_10_ (*p*-value) = 2 on the y-axis, and is represented by the horizontal dashed line. The line perpendicular to the x-axis represents a fold-change cutoff. **(B)** Subcellular localization of the identified proteins. **(C)** Subset of proteins identified in the Ca_v_β_2b_ nanoenvironments in cardiomyocytes, categorized according to their biological function. A complete list of proteins and their functions is provided in Supplementary Table S2. E-C coupling, excitation-contraction coupling.

We next identified the proteins encountered in the Ca_v_β_2b_ nano-environments using APEX2-mediated proximity biotinylation followed by streptavidin-mediated affinity purification and liquid chromatography-tandem mass spectrometry (LC-MS/MS). Three label-free independent experiments were performed using Ca_v_β_2b_-V5-APEX2-expressing cardiomyocytes treated with biotin-phenol in the presence or absence (negative control) of H_2_O_2_. It is important to note that some proteins located in the Ca_v_β_2b_ nanoenvironments can remain unidentified by this proteomics approach. Some peptides may not be detected by LC-MS/MS due to many factors, including inherent unsuitability for mass spectrometry, steric obstruction of biotin labeling or lack of suitable reactive moieties in the protein sequence (Paek et al., 2017). Nevertheless, 603 proteins having at least two peptides detected by LC-MS/MS were identified in the three experiments, and 440 of these proteins were consistently detected in each of their three replicas. In order to exclude from our evaluation the proteins detected in the negative control samples, we performed label-free quantitative (LFQ) analyses. The cutoff and p values that we used for the classification of proteins as proximal proteins separated enriched proteins from background binders. This analysis also eliminated candidates with either lower abundance in cardiomyocytes or less stable interaction with Ca_v_β_2b_-associated networks and enabled the enrichment of candidates from the Ca_v_β_2b_ nanoenvironments. With this final approach, 61 proteins were annotated as components of the Ca_v_β_2b_ nanoenvironments (Figure 2A; Supplementary Table S1).

Functional analyses, using information from the UniProtKB/Swiss-Prot and Pubmed databases and the DAVID Functional Annotation Tool (http://david.abcc.ncifcrf.gov) (Huang et al., 2009), revealed that most of the proteins identified as constituents of the Ca_v_β_2b_ nanoenvironments are cytosolic (35%), although plasma membrane (20%), nuclear (19%) and cytoskeleton-associated (19%) proteins were also detected (Figure 2B; Supplementary Table S2). The remaining constituents of the Ca_v_β_2b_ nanoenvironments were annotated as proteins from the sarcoplasmic reticulum (6%) or having an unknown subcellular localization. Based on their function, the 61 proteins were further grouped into 11 different categories: proteins involved in cellular trafficking (11; 18%), muscular contraction (8; 13%), sarcomere organization (7; 11%), protein translation (7; 11%), excitation-contraction coupling (6; 10%) and protein degradation (6; 10%). A subset and a full list of the identified proteins, including their main functions and annotation, are provided in Figure 2C and Supplementary Table S2, respectively.

### Ca_v_β_2b_ and the RyR2 are Part of a Macromolecular Complex in Cardiomyocytes

**FIGURE 3:**
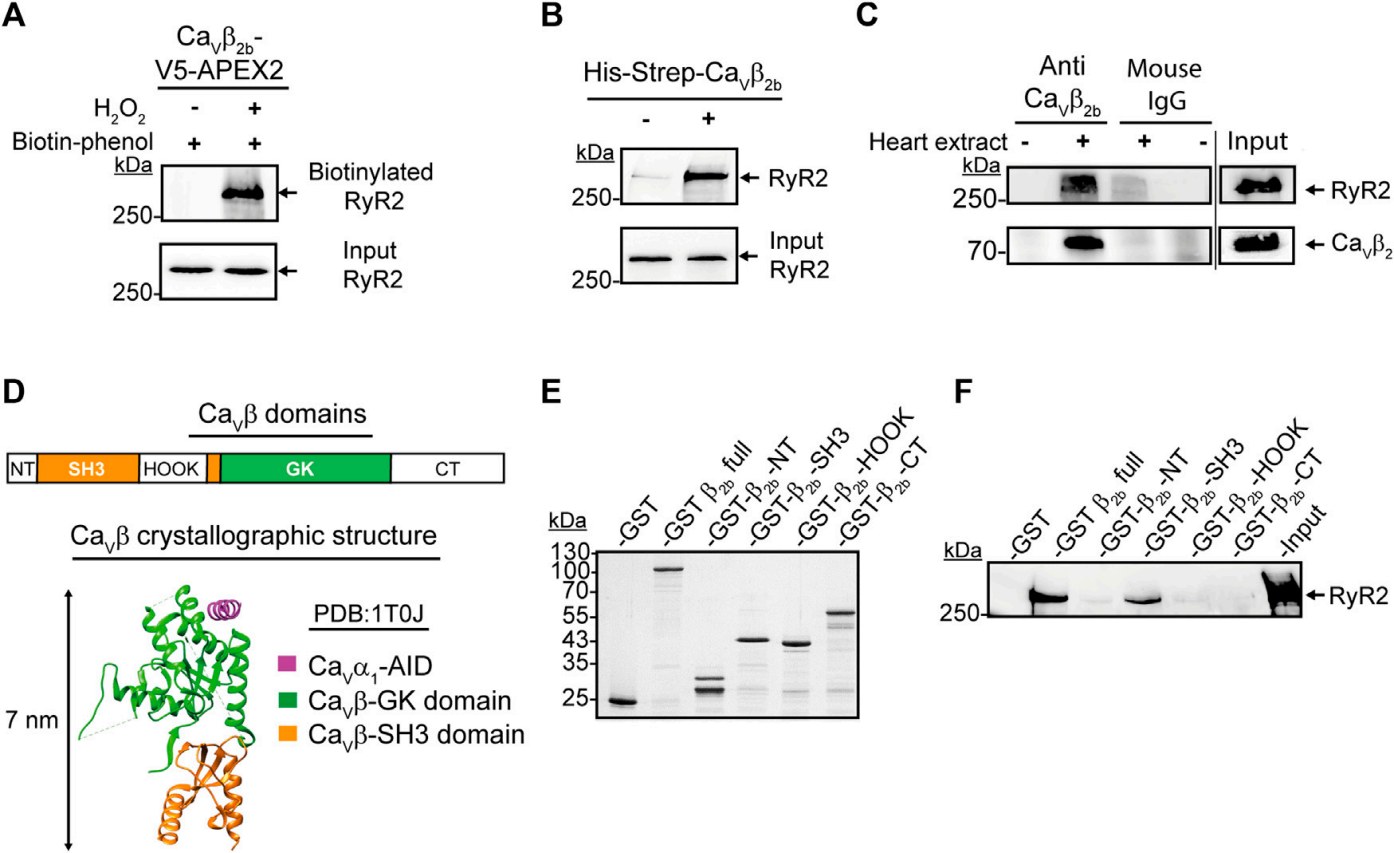
Interaction between Ca_v_β_2b_ and the RyR2. **(A)** Biotinylation of the RyR2 by Ca_v_β_2b_-V5-APEX2. Western blot analysis using an anti-RyR2 antibody after streptavidin-mediated enrichment of biotinylated proteins. **(B)** Pull-down assay of the RyR2 with the recombinant His-Strep-Ca_v_β_2b_. Mouse heart lysates were incubated with Strep-Tactin^®^-coated beads with or without the purified His-Strep-Ca_v_β_2b_ and the eluates were analyzed by western blot using an anti-RyR2 antibody. **(C)** Co-immunoprecipitation of the RyR2 with Ca_v_β_2b_ from mouse heart lysates. Ca_v_β_2b_ was immunoprecipitated using an anti-Ca_v_β_2b_ antibody and the co-precipitated RyR2 was analyzed by western blot. Normal mouse IgG was used as negative control. The upper and lower panels show a western blot using anti-RyR2 and a commercial anti-Ca_v_β_2_ antibodies, respectively. **(D)** Schematic diagram of Ca_v_β_2b_ domains: NT (N-terminus), SH3 (Src-homology 3 domain), HOOK, GK (guanylate kinase domain), CT (C-terminus) (i) and crystal structure (PDB:1T0J) (van Petegem et al., 2004) of the Ca_v_β in a complex with the AID of Ca_v_α_1_ (magenta), Ca_v_β-GK (green) and Ca_v_β-SH3 (orange) domains (ii) are shown. **(E)** Coomassie blue-stained SDS-PAGE gel with the purified recombinant proteins used in panel f (1 μg per lane). **(F)** Pull-down assay of the RyR2 with the purified recombinant Ca_v_β_2b_ domain proteins. Strep-Tactin^®^-coated beads coupled to the purified recombinant proteins were incubated with mouse heart lysates and the eluates were analyzed by western blot using an anti-RyR2 antibody. The GST and Ca_v_β_2_ full protein were used as negative and positive controls, respectively. All the data shown are representative of three independent experiments. AID, alpha interaction domain; RyR2, ryanodine receptor 2.

One of the proteins detected with the highest accuracy as a constituent of the Ca_v_β_2b_ nanoenvironments was the RyR2. In total, 77 peptides from this protein were detected by LC-MS/MS (Supplementary Table S1). The RyR2 was clearly detected in cardiomyocytes expressing Ca_v_β_2b_-V5-APEX2 and treated with biotin-phenol and H_2_O_2_, as shown by the western blot analysis after the streptavidin-mediated affinity purification of the cell lysates (Figure 3A). This result confirms the findings from LC-MS/MS and the close proximity between Ca_v_β_2b_ and the RyR2.

Since APEX2-mediated proximity biotinylation targets proteins located within 20 nm of the APEX2 molecule (Martell et al., 2012), the biotin labeling of one such protein does not necessarily imply an interaction with the APEX2-fused protein. In order to assess whether Ca_v_β_2b_ interacts with the RyR2 in cardiomyocytes, we performed pull-down assays using a recombinant Strep-tagged Ca_v_β_2b_ as bait and whole mouse heart extracts. Western blot analyses of the pull-down assay eluates revealed an interaction between Ca_v_β_2b_ and the RyR2 (Figure 3B). Moreover, co-immunoprecipitation assays using whole heart extracts and the anti-Ca_v_β_2b_ antibody corroborated the existence of a complex between Ca_v_β_2b_ and the RyR2 in living cardiomyocytes (Figure 3C).

### The SH3 Domain of Ca_v_β_2b_ Mediates the Interaction With the RyR2

Ca_v_β consists of five domains: NT, SH3, HOOK, GK, and CT (Figure 3Di). However, crystallographic and biochemical studies have demonstrated that only the GK domain interacts with the AID in Ca_v_α_1_ (van Petegem et al., 2004; Gonzalez-Gutierrez et al., 2008; Figure 3Dii). In order to identify the Ca_v_β_2b_ domains interacting with the RyR2, we expressed and purified from *E. coli* the different GST-Strep-tagged Ca_v_β domains (Figure 3E). Ca_v_β-GK was excluded from the analysis because this fusion protein aggregates into inclusion bodies when expressed in *E. coli*. Since aggregated proteins often lose their three-dimensional structure and are unable to properly interact with other proteins, we excluded the GK domain from the analysis. Moreover, Ca_v_β-GK strongly interacts with the Ca_v_α_1_ subunit, functioning as a regulator of the channel activity, and it is therefore unlikely to interact also with the RyR2 (Gonzalez-Gutierrez et al., 2008). Pull-down assays using whole heart extracts and Strep-Tactin^®^-coated beads coupled to the different GST-Strep-Ca_v_β_2b_ domains, demonstrated that only the Ca_v_β_2b_-SH3 domain interacts with the RyR2 (Figure 3F).

### Cellular Distribution of Ca_v_β_2b_

**FIGURE 4:**
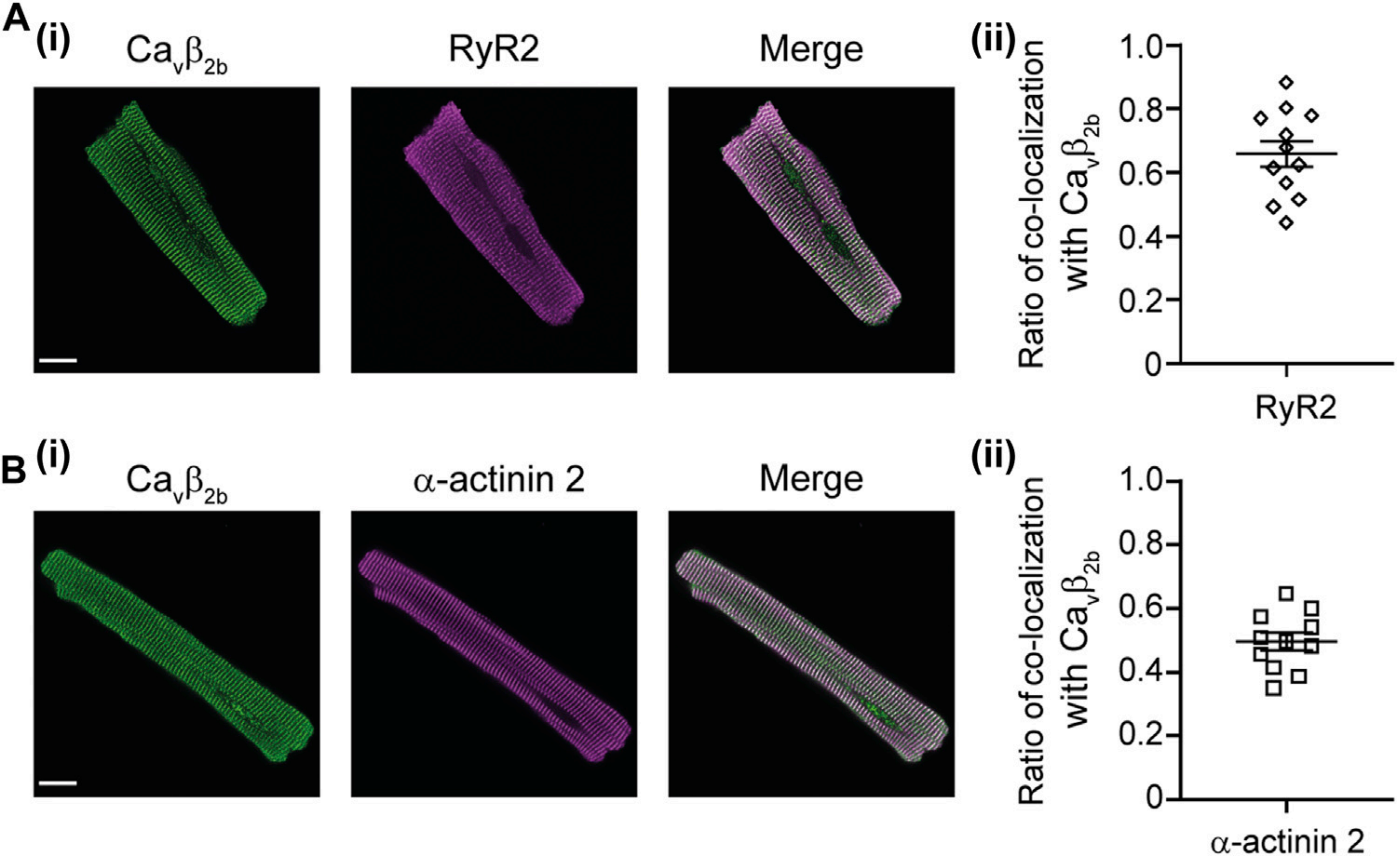
Confocal images of immunostained ARCs. **(A)** Confocal images of a representative ARC stained with antibodies recognizing Ca_v_β_2b_ (green) and RyR2 (magenta) **(i)** and scatter plot showing ratio of co-localization of Ca_v_β_2b_ with the RyR2 (*n* = 12 cells), (**ii**). **(B)** Confocal images of a representative ARC stained with antibodies recognizing Ca_v_β_2b_ (green) and α-actinin 2 (magenta) **(i)** and scatter plot showing ratio of co-localization of Ca_v_β_2b_ with α-actinin 2 (*n* = 11 cells), (**ii**). Scale bars represent 15 µm. ARCs, adult rat cardiomyocytes; RyR2, ryanodine receptor 2.

Confocal imaging showed a distribution pattern of Ca_v_β_2b_ that resembled the arrangement of t-tubules previously revealed with other markers (Wong et al., 2013; Hong and Shaw 2017; Figure 1Dii). To further study the relative distribution of Ca_v_β_2b_ and the RyR2, we performed co-localization analyses in isolated ARCs using confocal imaging (Figure 4Ai). We also visualized the cellular distribution of Ca_v_β_2b_ and α-actinin 2 (Figure 4Bi), a protein known to connect the cardiac t-tubule membrane with the sarcomere Z-lines (Hong and Shaw, 2017), and that was also detected in the nanoenvironments of Ca_v_β_2b_ by the LC-MS/MS analyses (Figure 2C; Supplementary Tables S1, S2). Quantification of the co-localization between Ca_v_β_2b_ and the RyR2 revealed that approximately 60% of Ca_v_β_2b_ co-localizes with the RyR2 clusters (Figure 4Aii). Moreover, around 50% of Ca_v_β_2b_ also co-localized with α-actinin 2 (Figure 4Bii).

### Functional Role of the Ca_v_β_2b_-RyR2 Interaction in Adult Rat Cardiomyocytes

**FIGURE 5:**
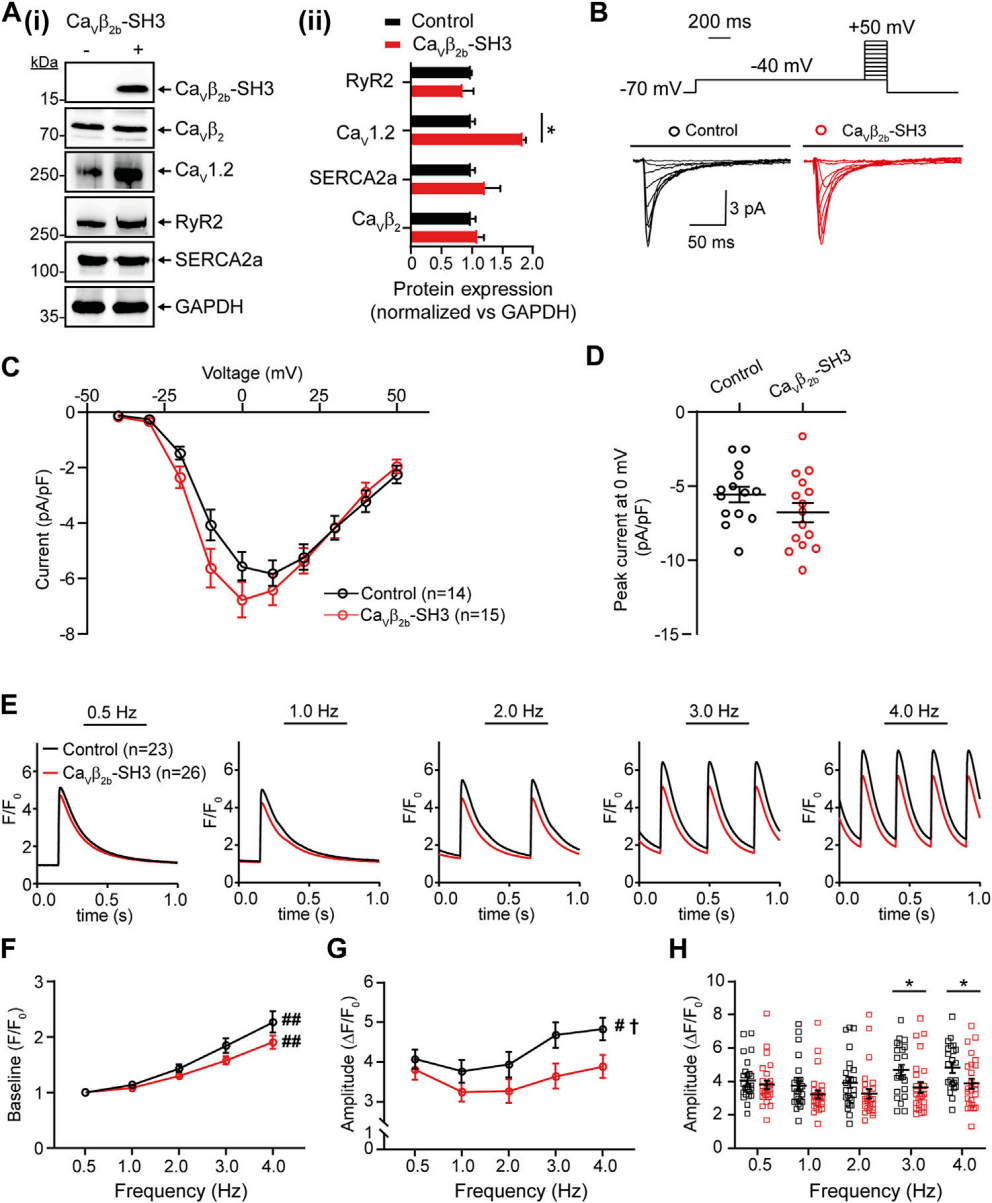
Endogenous L-type Ca^2+^ currents and Ca^2+^ transients measured at different pacing frequencies in ARCs expressing Ca_v_β_2b_-SH3. **(A)** Western blot analysis of the expression of Ca_v_β_2_, Ca_v_1.2, RyR2, SERCA2a, and GAPDH in ARCs after Ca_v_β_2b_-SH3 transduction **(i)** and bar plot of mean values of densitometry analyses from western blots **(ii)**. The expression of construct Ca_v_β_2b_-SH3, which contains a V5-tag at the C-terminus, was detected using an anti-V5 antibody. The protein expressions in the right panel were normalized vs GAPDH. **(B)** Voltage step protocol and representative whole-cell endogenous L-type Ca^2+^ current traces of control (black) and Ca_v_β_2b_-SH3-expressing (red) cardiomyocytes. **(C)** Current-voltage curves of L-type Ca^2+^ currents of control (black) and Ca_v_β_2b_-SH3-expressing (red) cardiomyocytes. **(D)** Scatter plot showing peak L-type Ca^2+^ currents at 0 mV in control (black) and Ca_v_β_2b_-SH3-expressing (red) cardiomyocytes. **(E)** Representative fluorescence traces of Ca^2+^ transients in control (black) and Ca_v_β_2b_-SH3-expressing (red) cardiomyocytes stimulated at pacing frequencies of 0.5, 1.0, 2.0, 3.0, and 4.0 Hz. **(F)** Diastolic (baseline) Ca^2+^ concentration at the different pacing frequencies. **(G)** Ca^2+^ transient amplitudes after baseline subtraction at the different pacing frequencies. **(H)** Scatter plot showing the Ca^2+^ transient amplitudes for each cell after baseline subtraction at the different pacing frequencies. Data are presented as mean ± SEM; ^#^ *p* < 0.05 between frequencies (one-way ANOVA); ^##^ *p* < 0.01 between frequencies (one-way ANOVA); ^†^ *p* < 0.05 between control and Ca_v_β_2b_-SH3-expressing ARCs (two-way ANOVA); *p < 0.05 between groups (unpaired *t*-test). The cells measured in the electrophysiological and Ca^2+^ transient analyses were from three different ARCs preparations. The numbers of cells per group measured in the electrophysiological experiments were: control, *n* = 14 and Ca_v_β_2b_-SH3-expressing cardiomyocytes, *n* = 15. For Ca^2+^ transient analysis were measured 23 and 26 cells from control and Ca_v_β_2b_-SH3-expressing, respectively. ARCs, adult rat cardiomyocytes; RyR2, ryanodine receptor 2; SERCA2a, sarcoplasmic reticulum-Ca^2+^ ATPase 2a.

We hypothesized that if the Ca_v_β_2b_-RyR2 interaction has a physiological role in linking LTCCs and RyR2 in cardiomyocytes, the overexpression of the SH3 domain of Ca_v_β_2b_ (Ca_v_β_2b_-SH3), which does not interact with Ca_v_α_1_ (van Petegem et al., 2004; Gonzalez-Gutierrez et al., 2007), could disrupt endogenous LTCC-RyR2 complexes and therefore affect CICR in these cells. We expressed a V5-tagged Ca_v_β_2b_-SH3 construct in ARCs using adenoviruses. Ca_v_β_2b_-SH3 expression was clearly detected by western blot in the cells transduced with this construct and did not affect the expression of endogenous Ca_v_β_2b_, RyR2 or SR-Ca^2+^ ATPase 2a (SERCA2a) (Figure 5A). In contrast, Ca_v_β_2b_-SH3 expression promoted an increase in the expression of Ca_v_1.2 (Figure 5A). In accordance, L-type Ca^2+^ currents from Ca_v_β_2b_-SH3 expressing cardiomyocytes were slightly larger, but not statistically different (*p* = 0.34), than those from control cells and the IV-curve was slightly shifted to more negative potentials (Figures 5B–D). Furthermore, no changes in the channel inactivation rate were observed in control cells as compared to Ca_v_β_2b_-SH3 expressing cardiomyocytes (Supplementary Figure S1).

To evaluate the effect of Ca_v_β_2b_-SH3 expression on CICR, intracellular Ca^2+^ transients were measured using the fluorescent calcium indicator Fluo-4AM at pacing frequencies between 0.5 and 4.0 Hz (Figure 5E). A rise in the diastolic (baseline) Ca^2+^ levels was observed in control and Ca_v_β_2b_-SH3-expressing cardiomyocytes when the stimulation frequencies increased, but no significant differences were observed between the two groups (Figure 5F). A pacing frequency-dependent increase in the amplitude of the Ca^2+^ transients was observed in control cells (Figure 5G). In contrast, this effect was completely absent in Ca_v_β_2b_-SH3-expressing cardiomyocytes (Figure 5G). The amplitudes of the Ca^2+^ transients measured at low pacing frequencies (0.5–2.0 Hz) were similar in control and Ca_v_β_2b_-SH3-expressing cardiomyocytes (Figure 5H). However, Ca_v_β_2b_-SH3-expressing cardiomyocytes displayed a significant decrease (∼25%) in the amplitude of their Ca^2+^ transients measured at 3.0 and 4.0 Hz as compared to controls (Figure 5H). The magnitude of SR-stored Ca^2+^, assessed by the amplitude of caffeine-induced Ca^2+^ release, or the rate of cytoplasmic Ca^2+^ removal through SERCA2a and the Na^+^/Ca^2+^ exchanger did not differ between control and Ca_v_β_2b_-SH3-expressing cardiomyocytes (Supplementary Figure S2).

## Discussion

A previous study identified 207 proteins as constituents of the nanoenvironments of the P/Q-, N- and R-type voltage-gated calcium channels in rat brain (Müller et al., 2010). During the preparation of the present manuscript, a similar study using transgenic mice overexpressing in the heart Ca_v_β_2b_ fused to APEX2, identified diverse proteins in the nanoenvironments of LTCCs (Liu et al., 2020). Here, using APEX2-mediated proximity biotinylation, we identified 61 proteins in the nanoenvironments of Ca_v_β_2b_ in cardiomyocytes. Most of these proteins have not previously been reported to be located in close proximity to Ca_v_β_2_ or LTCCs. Many of them are involved in Ca^2+^-dependent processes like excitation-contraction coupling, cardiac contraction and cellular trafficking (Figure 2C; Supplementary Table S2). However, it is worth mentioning that not all the proteins identified as part of the Ca_v_β_2b_ nanoenvironments were expected to localize close to the LTCCs. Ca_v_β is a scaffolding modular protein that could interact in parallel with diverse proteins through its SH3 and GK domains. In accordance, diverse LTCC-independent functions have been reported for Ca_v_β in different cell types (Berggren et al., 2004; Ebert et al., 2008; Jha et al., 2009; Miranda-Laferte et al., 2011; Hofmann et al., 2015; Stolting et al., 2015). Our proteomics analyses confirmed the close proximity between Ca_v_β and some of its known non-LTCC-related binding partners such as AHNAK (Pankonien et al., 2012) and protein phosphatase 2A (Tadmouri et al., 2012). Moreover, they revealed that Ca_v_β_2b_ is also present in the nanoenvironments of some Z-line-associated proteins like α-actinin 2, nebulin-related-anchoring protein, LIM-binding protein 3 and vinculin (Figure 2C; Supplementary Table S2). These proteins play an important role in the structural organization of cardiomyocytes by anchoring actin filaments to the Z-lines (Frank and Frey, 2011). Therefore, the close proximity between Ca_v_β_2b_ and these Z-line-associated proteins, together with the reported interaction of Ca_v_β_2b_ with F-actin (Stolting et al., 2015), strongly suggest that Ca_v_β_2b_ could play a role as a scaffold, participating in the organization of Z-line protein complexes.

One of the proteins found in the nanoenvironments of Ca_v_β_2b_ was the RyR2. Due to the high physiological relevance of LTCCs and RyR2 for excitation-contraction coupling in cardiomyocytes, we specifically focused on the functional role of the close proximity between Ca_v_β_2b_ and the RyR2. In skeletal muscle cells, Ca_v_α_1a_ mediates the interaction between LTCCs and the RyR1 which is necessary to transduce plasma membrane depolarization into the opening of the RyR1 in the SR membrane and to trigger the release of Ca^2+^ ions from this compartment (Cheng et al., 2005). The interaction between LTCCs and the RyR1 has also been reported to occur in the brain (Mouton et al., 2001). In contrast, an interaction between these two molecules is apparently not necessary in cardiomyocytes, since the influx of Ca^2+^ through the LTCCs leads to the opening of the RyR2 and the release of large amounts of Ca^2+^ ions from the SR (Eisner et al., 2017; Bers, 2018). However, our proteomics and biochemical analyses demonstrated that Ca_v_β_2b_ and the RyR2 do interact in cardiomyocytes. A similar result was observed in embryonic fibroblasts, where Ca_v_β_3_ has been reported to physically interact with the IP3-receptor, a protein also responsible for the release of Ca^2+^ ions from the SR (Belkacemi et al., 2018). Moreover, our confocal imaging showed that Ca_v_β_2b_ is widely distributed throughout the t-tubules and co-localizes with the RyR2 at the dyads (Figures 4A,B). Since Ca_v_β_2b_ is strongly associated with LTCCs through its GK domain (van Petegem et al., 2004; Gonzalez-Gutierrez et al., 2008) and its SH3 domain interacts with RyR2, our results indicate that probably Ca_v_β_2b_ is mediating a macromolecular complex between LTCCs and RyR2 in adult cardiomyocytes.

**FIGURE 6:**
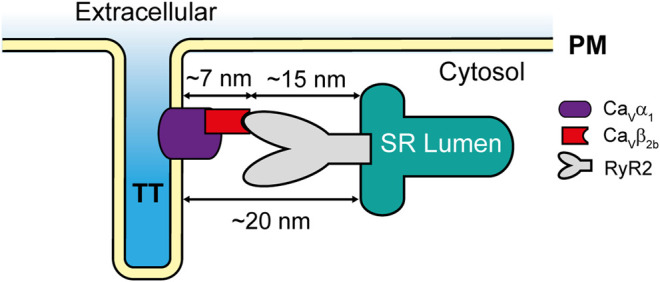
Schematic representation of the LTCC-RyR2 interaction in adult rat cardiomyocytes. PM, plasma membrane; RyR2, ryanodine receptor 2; SR, sarcoplasmic reticulum; TT, t-tubules.

The localization of LTCCs and the RyR2 in different cellular compartments (t-tubules and SR, respectively) raises the question of whether LTCCs and RyR2 can interact. According to the published cryo-electron microscopy (cryo-EM) structure of an LTCC complex (Wu et al., 2015), the Ca_v_β_2_ subunit projects ∼7 nm away from the channel and into the cytosol. Additionally, a cryo-EM structure of the RyR2 (Peng et al., 2016) showed that its big cytosolic domain protrudes ∼15 nm toward the cytosolic space surrounding the SR. Therefore, since the gap between the t-tubules and the SR cisternae is around 12–20 nm at the dyads (Shaw and Colecraft, 2013), it allows Ca_v_β_2_ to potentially mediate the docking of some LTCCs at the RyR2 clusters in cardiomyocytes (Figure 6). Together, our results suggest that the Ca_v_β_2b_ subunit participates in the association of LTCCs with the RyR2 at the dyads. These complexes between LTCCs and RyR2 could increase the probability that entering Ca^2+^ ions may find their binding site on the RyR2, thus reducing the number of ions required for RyR2 activation and increasing the efficiency of the CICR mechanism in cardiomyocytes.

Our biochemical studies demonstrated that the interaction between Ca_v_β_2b_ and the RyR2 is mediated by the SH3 domain of Ca_v_β_2b_. Hence, we rationalized that overexpression of this domain in ARCs would disturb the LTCC-RyR2 complexes and thereby allow us to study the functional relevance of this interaction for CICR. Unfortunately, our imaging and biochemical methods could not evaluate the extent of uncoupling of the complexes between endogenous Ca_v_β_2b_ and RyR2 after Ca_v_β_2b_-SH3 overexpression. While confocal imaging lacks the appropriate resolution, our co-immunoprecipitation experiments using lysates from ARCs failed due to the low protein yield after cellular lysis.

In humans, the frequency-dependent upregulation of CICR in the cardiac muscle cells is an intrinsic regulatory mechanism leading to a rise in the cardiac contractile force at high pacing frequencies. This allows an adequate cardiac output due to a faster filling of the heart chambers during the shorter diastolic periods occurring at high beating rates (Kushnir et al., 2010). However in rats, analyses of the force-frequency relationships have provided contradictory results, with positive (Layland and Kentish, 1999; Dibb et al., 2007) or negative (Bouchard and Bose, 1989) FFR being reported. Central to this contradiction are the conditions under which experiments are performed, especially the pacing frequency, the temperature and the extracellular calcium concentration. In accordance with another study (Gattoni et al., 2016), our results show a flat FFR at stimulation frequencies 0.5–2.0 Hz, whereas at 3.0–4.0 Hz FFR was positive.

We have expressed Ca_v_β_2b_-SH3 in ARCs in order to disrupt the possible Ca_v_β_2b_-mediated interaction between LTCCs and the RyR2. Interestingly, the expression of Ca_v_β_2b_-SH3 in ARCs produced an upregulation in Ca_v_1.2 expression but not an increase in L-type Ca^2+^ currents. In accordance, we did not detect differences in the amplitude of the Ca^2+^ transients between control and Ca_v_β_2b_-SH3-expressing cardiomyocytes measured at low pacing frequencies (0.5–2.0 Hz). Moreover, the lack of differences between calcium transients from control and Ca_v_β_2b_-SH3-expressing cardiomyocytes at low pacing frequencies suggests that the Ca_v_β_2b_-mediated interaction between LTCCs and the RyR2 does not play a critical role in CICR at low myocyte beating rates. However, high pacing frequencies (3.0–4.0 Hz) significantly increased the amplitude of the Ca^2+^ transients in control ARCs, but this response was fully absent in Ca_v_β_2b_-SH3 expressing ARCs. Since Ca_v_β_2b_-SH3 expression does not affect LTCC inactivation, this absence of pacing frequency-dependent increase of CICR in Ca_v_β_2b_-SH3 expressing ARCs, strongly suggests that the interaction between LTCCs and the RyR2 mediated by Ca_v_β_2b_ is necessary to upregulate CICR at high pacing frequencies.

Overall, in our work we mapped the protein interaction network of Ca_v_β_2b_ in rodent hearts and demonstrated that an interaction occurs between this protein and one of its identified partners, the RyR2, mediated by the Ca_v_β_2b_-SH3 domain. Further characterization of this interaction will be necessary in order to determine the Ca_v_β_2b_-binding site on the RyR2.

We also took the first steps to study the function of the interaction between Ca_v_β_2b_ and the RyR2, which seems to have an important role in CICR at high cardiomyocyte beating rates. We assumed that the overexpression of the Ca_v_β_2b_-SH3 domain would displace the endogenous Ca_v_β_2b_ from its interaction with the RyR2. However, this premise was not demonstrated in our work and is its main limitation. Moreover, it is worth mentioning that the use of the Ca_v_β_2b_-SH3 domain to displace native Ca_v_β_2b_ from its interaction with the RyR2 could bring about the disruption of the interaction of Ca_v_β_2b_ with other proteins. So far, only two proteins, dynamin and actin, have been reported to interact with Ca_v_β_2_ through its SH3 domain (Gonzalez-Gutierrez et al., 2007; Stolting et al., 2015). Dynamin regulates the endocytosis of Cav1.2 and actin seems to participate in the traffic of the channel to the cell surface. Although the displacement of Ca_v_β_2_ from its interaction with these two partners would not explain the fact that the changes in Ca^2+^ transients only occur at high stimulation frequencies, we cannot rule out the possibility that other proteins are affected.

## Data Availability

The mass spectrometry proteomics data have been deposited to the ProteomeXchange Consortium via the PRIDE partner repository with the dataset identifier PXD021375 and 10.6019/PXD021375.
