# Supplementary material for: Nanoenviroments of the β-Subunit of L-Type Voltage-Gated Calcium Channels in Adult Cardiomyocytes
Source: Front Cell Dev Biol. 2022 Jan 3;9:724778. doi: 10.3389/fcell.2021.724778 (PMC8762238; doi:10.3389/fcell.2021.724778)
Supplement: Supplementary file 1 [file Table2.pdf]

Supplementary Table S2. Annotation of the proteins identified in the nanoenvironments of $Ca_v\beta_{2b}$

Protein ID	Protein name	Subcellular localization	Biological function
A0A096MJ01	LIM domain-binding 3	Cytoskeleton	Sarcomere organization
A0A096MJI9	Na^+/K^+ transporting ATPase subunit beta	Membrane	Ion transport
A0A0G2JSH9	Peroxiredoxin-2	Cytosol	Cellular response to oxidative stress
A0A0G2JSU4	N-myc downstream regulated gene 2	Nucleus	Unknown
A0A0G2JU96	AHNAK nucleoprotein	Cytosol, membrane, nucleus	Excitation-contraction coupling, sarcomere organization, cellular trafficking
A0A0G2JUC7	Dynactin subunit 2	Cytoskeleton	Cellular trafficking
A0A0G2JWE1	Myosin light polypeptide 6	Cytosol	Unknown
A0A0G2K1J5	Plectin	Cytoskeleton	Cell-cell adhesion
A0A0G2K2T1	Taxilin beta	Cytosol	Cellular trafficking
A0A0G2K5P5	Myomesin 1	Cytoskeleton	Muscle contraction
A0A0G2K6S9	Myosin-11	Cytoskeleton	Muscle contraction
A0A0G2K7B6	Dysferlin	Cytoskeleton, membrane	Plasma membrane repair
R9PXU6	Vinculin	Cytoskeleton	Sarcomere organization, cell-cell adhesion
A0A0H2UHR7	Filamin-C	Cytoskeleton	Unknown
A0JPQ4	Tripartite motif-containing protein 72	Membrane	Plasma membrane repair
B0LPN4	Ryanodine receptor 2	Sarcoplasmic reticulum	Excitation-contraction coupling, ion transport
B1PRL5	Caveolae-associated protein 4	Cytosol, membrane	Sarcomere organization
D3Z802	Nebulin-related-anchoring protein	Cytoskeleton	Sarcomere organization
D3ZCV0	α-actinin 2	Cytosol, membrane	Sarcomere organization
D4AC36	Eukaryotic translation initiation factor 3 subunit F	Cytosol	Protein translation
E9PT87	Myosin light chain kinase 3	Cytosol, cytoskeleton	Muscle contraction
F1M779	Clathrin heavy chain	Membrane	Cellular trafficking
Q62812	Myosin-9	Cytoskeleton	Muscle contraction
G3V7K1	Myomesin 2	Cytoskeleton	Muscle contraction

G3V7L6	26S proteasome regulatory subunit 7	Cytosol	Protein degradation
G3V9N0	Polyadenylate-binding protein	Cytosol	Protein translation
M0R9L0	Nascent polypeptide-associated complex subunit alpha	Unknown	Unknown
M0R9X8	Cytoplasmic dynein 1 heavy chain 1	Cytosol, membrane, cytoskeleton	Cellular trafficking
O35303	Dynamin-1-like protein	Membrane, cytosol	Cellular trafficking
O35878	Heat shock protein beta-2	Cytosol, nucleus	Protein folding
P05197	Elongation factor 2	Cytosol, nucleus	Protein translation
P08733	Myosin regulatory light chain 2	Cytoskeleton	Muscle contraction
P09456	cAMP-dependent protein kinase type I-alpha regulatory subunit	Cytosol, membrane	Excitation-contraction coupling, Ca^{2+} signaling
P0DMW1	Heat shock 70 kDa protein 1B	Cytosol, cytoskeleton	Protein folding
P11232	Thioredoxin	Cytosol, nucleus	Cellular response to oxidative stress
P19804	Nucleoside diphosphate kinase B	Cytosol, nucleus	Unknown
P36201	Cysteine-rich protein 2	Cytosol, nucleus	Unknown
P46462	Transitional endoplasmic reticulum ATPase	Cytosol, nucleus, sarcoplasmic reticulum	Protein degradation, cellular trafficking
P56741	Myosin-binding protein C, cardiac-type	Cytoskeleton	Sarcomere organization, muscle contraction, cell-cell adhesion
Q5D059	Heterogeneous nuclear ribonucleoprotein K	Nucleus	Transcription regulator
P61983	14-3-3 protein gamma	Cytosol, membrane	Unknown
P63018	Heat shock cognate 71 kDa protein	Membrane, nucleus	Protein folding
Q1JU68	Eukaryotic translation initiation factor 3 subunit A	Cytosol, nucleus	Protein translation
Q2PQA9	Kinesin-1 heavy chain	Cytoskeleton	Cellular trafficking
Q2PS20	Junctophilin-2	Membrane, sarcoplasmic reticulum	Sarcomere organization, excitation-contraction coupling

Q4FZT9	26S proteasome non-ATPase regulatory subunit 2	Cytosol	Protein degradation
Q4G061	Eukaryotic translation initiation factor 3 subunit B	Cytosol, nucleus	Protein translation
Q4PP99	Cardiac troponin C	Cytoskeleton	Muscle contraction
Q5U2U8	Bcl2-associated athanogene 3	Cytosol	Protein folding
Q5U300	Ubiquitin-like modifier-activating enzyme 1	Cytosol	Protein degradation
Q5XI34	Protein phosphatase 2 (formerly 2A), regulatory subunit A (PR 65)	Cytosol, nucleus	Excitation-contraction coupling, Ca^{2+} signaling, protein translation
Q6P6U2	26S proteasome regulatory subunit 6A	Cytosol	Protein degradation
Q68FR6	Elongation factor 1-gamma	Cytosol, nucleus	Protein translation
Q68FR9	Elongation factor 1-delta	Cytosol, nucleus	Protein translation
Q68FS2	COP9 signalosome complex subunit 4	Cytosol, nucleus	Protein degradation
P48037	Annexin A6	Membrane, nucleus	Cellular trafficking
Q6P9V7	Proteasome activator subunit 1	Cytosol	Protein degradation
Q8R3Z7	EH-domain-containing 4	Membrane, sarcoplasmic reticulum	Cell-cell adhesion
Q9EPX0	Heat shock protein beta-8	Cytosol, nucleus	Protein folding
Q9Z269	Vesicle-associated membrane protein-associated protein B	Membrane, sarcoplasmic reticulum	Cellular trafficking
Q9Z270	Vesicle-associated membrane protein-associated protein A	Membrane, sarcoplasmic reticulum	Cellular trafficking
P22002[a]	Voltage-dependent L-type calcium channel subunit alpha-1C	Membrane	Excitation-contraction coupling, ion transport

[a] Identified by western blot
