# Supplementary material for: Nanoenviroments of the β-Subunit of L-Type Voltage-Gated Calcium Channels in Adult Cardiomyocytes
Source: Front Cell Dev Biol. 2022 Jan 3;9:724778. doi: 10.3389/fcell.2021.724778 (PMC8762238; doi:10.3389/fcell.2021.724778)
Supplement: Supplementary file 2 [file DataSheet1.pdf]

Supplementary Figures

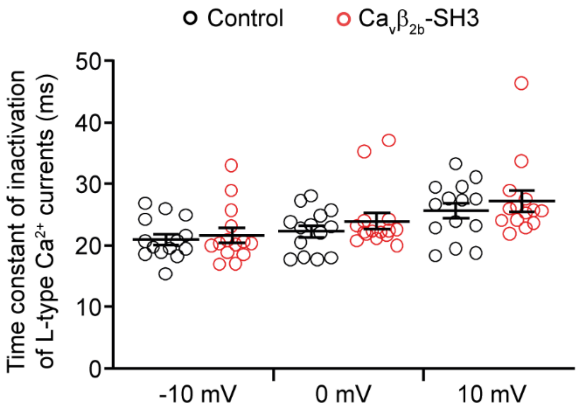

Supplementary Figure S1. Time constant of inactivation of L-type Ca^{2+} currents in adult rat cardiomyocytes expressing Ca$_v$β$_{2b}$-SH3. Scatter plot showing the time constant (tau) of inactivation of L-type Ca^{2+} current at -10, 0 and 10 mV of control (black, n=14) and Ca$_v$β$_{2b}$-SH3-expressing (red, n=15) cardiomyocytes. Data are presented as mean ± SEM.

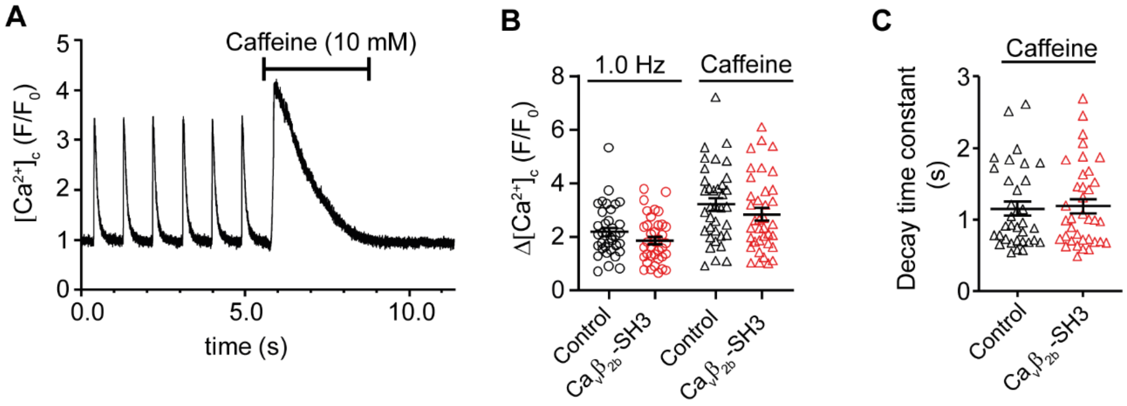

Supplementary Figure S2. **Sarcoplasmic reticulum Ca^{2+} content and cytosolic Ca^{2+} removal in cardiomyocytes overexpressing the SH3 domain of Ca$_v$β$_{2b}$.** Adult rat cardiomyocytes were paced at 1.0 Hz for 1 minute to establish steady-state contractions. Then, the pacing was paused and the release of the total SR Ca^{2+} content was induced by fast perfusion with normal Tyrode's solution supplemented with 1.8 mM Ca^{2+} and 10 mM caffeine. (**A**) Representative fluorescence tracing during the 1.0 Hz pacing and Ca^{2+} release induced by application of caffeine. (**B**) Ca^{2+} transient amplitude at 1.0 Hz and SR Ca^{2+} content assessed by caffeine-induced Ca^{2+} release; n=35 control cells, n=37 Ca$_v$β$_{2b}$-SH3-expressing cells. (**C**) Decay time constant; n = 34 control cells, n=36 Ca$_v$β$_{2b}$-SH3-expressing cells. Data are presented as mean ± SEM.
